# The impact of SARS-CoV-2 VOCs on clinical outcomes: an overview of reviews

**DOI:** 10.3389/fmed.2025.1624459

**Published:** 2025-08-14

**Authors:** Federico Fama, Rebecca Fattore, Paolo Raimondo, Fabio Brivio, Darcy Holmes, Toussaint Muheberimana, Tarek Nayfeh, Alessandra Bandera, Andrea Gori, Matteo Passerini, Marta Colaneri

**Affiliations:** ^1^Department of Infectious Diseases, Luigi Sacco University Hospital, Milan, Italy; ^2^Centre for Multidisciplinary Research in Health Science (MACH), University of Milan, Milan, Italy; ^3^Infectious Diseases Unit, Fondazione IRCCS Ca' Granda Ospedale Maggiore Policlinico, Milan, Italy; ^4^Evidence-Based Practice Center, Mayo Clinic, Rochester, MN, United States; ^5^Department of Pathophysiology and Transplantation, University of Milan, Milan, Italy; ^6^Department of Biomedical and Clinical Sciences, University of Milan, Milan, Italy

**Keywords:** SARS-CoV-2, Omicron, Delta, VOCs, systematic reviews, COVID-19

## Abstract

**Background:**

Synthesizing data from existing literature is crucial for validating the robustness of associations, assessing data quality, and forming recommendations, especially given the vast amount of information available on SARS-CoV-2. This study aims to conduct an overview of reviews to evaluate the strength and validity of associations between VOCs and specific clinical outcomes in COVID-19 patients.

**Methods:**

An overview of reviews according to the principles of PRIOR protocol was performed searching multiple databases in January 2024 and an updated search was conducted in MEDLINE database in June 2025. Peer reviewed systematic reviews considering two or more VOCs and reporting on clinical outcomes such as mortality, hospitalization, severe disease, admission to ICU, and mechanical ventilation were included. Data on study population and measures of association between clinical outcome and VOCs were considered. The quality of the studies was assessed through the AMSTAR-2 tool. Effect sizes and confidence intervals for each association between VOCs and clinical outcomes were reported. Subgroup analyses were performed where feasible. A citation matrix was used to assess the overlap between the included systematic reviews.

**Results:**

Twelve studies were included in the review, with a total of 24 comparisons, primarily between Omicron and Delta variants (19/24). Omicron was consistently associated with better clinical outcomes compared to Delta. The confidence in the results of 10/12 studies was rated critically low. The overlap between the included reviews was minimal, with 10% having significant overlap (>15%).

**Conclusion:**

Our overview of reviews shows the lower hazard on human health of the Omicron compared to Delta variant. However, the quality of the reviews included was generally low, prompting the need for more rigorous systematic reviews.

**Systematic review registration:**

This overview of reviews was registered in PROSPERO, CRD42024500841; https://www.crd.york.ac.uk/PROSPERO/display_record.php?ID=CRD42024500841.

## Background

The severe acute respiratory syndrome coronavirus 2 (SARS-CoV-2) pandemic represents one of the deadliest outbreaks of this century, causing an unprecedented global health crisis. Since its emergence in December 2019, the novel virus has caused approximately 778 million cases and at least 7.1 million deaths (1).

Following the first viral strain detected in Wuhan in 2019, multiple mutations have led to the emergence of variants of concern (VOCs) that circulated all over the world, changing the trajectory of the pandemic in terms of infections rates, severity of disease and death (2). Since then, the variants of concern have remained within the Omicron lineage, with JN.1 and KS.1 currently being the most relevant sublineages. These Omicron subvariants continue to drive new waves of infection, and their identification and tracking remain central to ongoing surveillance efforts, as highlighted by recent molecular and epidemiological studies (3, 4). On the other hand, earlier VOCs—Alpha, Beta, Gamma, and Delta—are no longer in significant circulation (3, 5). Together with the development and distribution of COVID-19 (coronavirus disease 2019) vaccines, a pivotal moment was the emergence of the Delta variant (B.1.617.2) noted for its high transmissibility and greater virulence than its predecessors. This VOC prevailed over other variants from late 2020 to early 2021 causing a significant spike in COVID-19 mortality. Another turning point was the subsequent emergence of the Omicron (B.1.1.529) and its subvariants, which are much more transmissible but less virulent than Delta (6).

Given the substantial and heterogeneous amount of evidence for clinical outcome associated with different VOCs, it is critical to understand how these variants impact COVID-19 clinical outcomes. This study aims to analyze systematic reviews, through an overview of reviews, that report on COVID-19 clinical outcomes stratified by VOCs.

## Methods

### Data sources and search strategy

A comprehensive literature search across several databases was conducted on January 18, 2024, with no language restrictions. The databases searched included Ovid MEDLINE 1946 to Present and Epub Ahead of Print, Ovid Embase (1974+), Web of Science Core Collection via Clarivate Analytics (1975+), and Scopus via Elsevier (1788+). Animal studies were excluded. The date limits were set between December 2022 and January 2024; an updated search was conducted in the MEDLINE database on June 10, 2025. The search strings were designed with a medical librarian with inputs from the study investigators. Controlled vocabulary terms were supplemented with keywords. The search strategy is available in the Supplementary File S1.

### Systematic review selection and data extraction

An overview of reviews was conducted using multiple databases in January 2024, in accordance with the principles and definitions to Preferred Reporting Items for Overviews of Reviews (PRIOR); the protocol was prospectively registered with the PROSPERO database (CRD42024500841) (7).

The following inclusion criteria were applied: (i) peer-reviewed systematic reviews with or without meta-analysis, (ii) with data on at least two VOCs, and (iii) with one clinical outcome of interest associated with one or more VOCs. Clinical outcomes under evaluation included: mortality, hospitalization, intensive care unit (ICU) admission, invasive ventilation, and severe disease. Exclusion criteria were: (i) systematic reviews focusing on infants and subjects under 18 years of age, (ii) systematic reviews with insufficient details to extract data on the total number of patients included, (iii) systematic reviews without an effect size of association between the clinical outcome and the VOCs, and (iv) narrative reviews or reviews conducted without systematic methodology. Although we initially intended to investigate any clinical outcome, we subsequently decided to focus solely on key outcomes; this decision distinguishes this overview of reviews from the original PROSPERO protocol.

Citations were managed using a systematic review software (Covidence), and duplicates were removed. All titles and abstracts were independently screened by at least two of the three reviewers (FB, RF, and PR), followed by a full-text review of the selected articles conducted by the same reviewers. Discrepancies were resolved through discussion with a fourth reviewer (FF). Data extraction was performed independently by at least two reviewers out of four (FB, FF, RF, and PR) with discrepancies resolved with another reviewer (MP). The following data were collected: (i) systematic reviews’ information: year of publication, source databases, and last date of literature search, (ii) included studies and patients’ information: inclusion criteria, number of studies included, number of patients included, (iii) outcomes’ information: VOCs considered, clinical outcomes considered, adjustments done, subgroup analyses, and effect estimates odds ratio (OR), risk ratio (RR) and hazard ratio (HR) with 95% confidence intervals (CIs).

### Confidence in the results assessment

The quality of included systematic reviews was assessed using the A MeaSurement Tool to Assess systematic Reviews 2 (AMSTAR-2). This tool consists of 16 items presented as questions, seven of which are considered critical. The overall confidence in the results of the reviews is rated as follows: (i) high, no or one non-critical weakness; (ii) moderate, more than one non-critical weakness; (iii) low, one critical flaw with or without non-critical weakness; (iv) critically low, more than one critical flaw with or without non-critical weakness (8). The AMSTAR-2 was filed independently by two of four authors (FB, RF, PR, and FF) and discrepancies resolved through discussion with another reviewer (MP).

### Data analysis

We reported effect size measures with the corresponding 95% CI for each association between VOCs and the clinical outcome of interest. For the studies that directly compared two or more VOCs, the effect size measures considered included HR, OR and RR. We also planned to report the effect size measures for any subgroups analyzed within the studies.

A citation matrix visually organizing systematic reviews was constructed to assess their overlap using the Graphical Representation of Overlap for OVErviews (GROOVE) tool. This matrix quantifies overlap through the Corrected Covered Area (CCA), which is classified as very high (>15%), high (11–15%), moderate (6–10%), or slight (0–5%). The CCA is a validated method for measuring overlap between reviews, aiding in decision-making on managing overlap (9).

## Results

**Figure 1:**
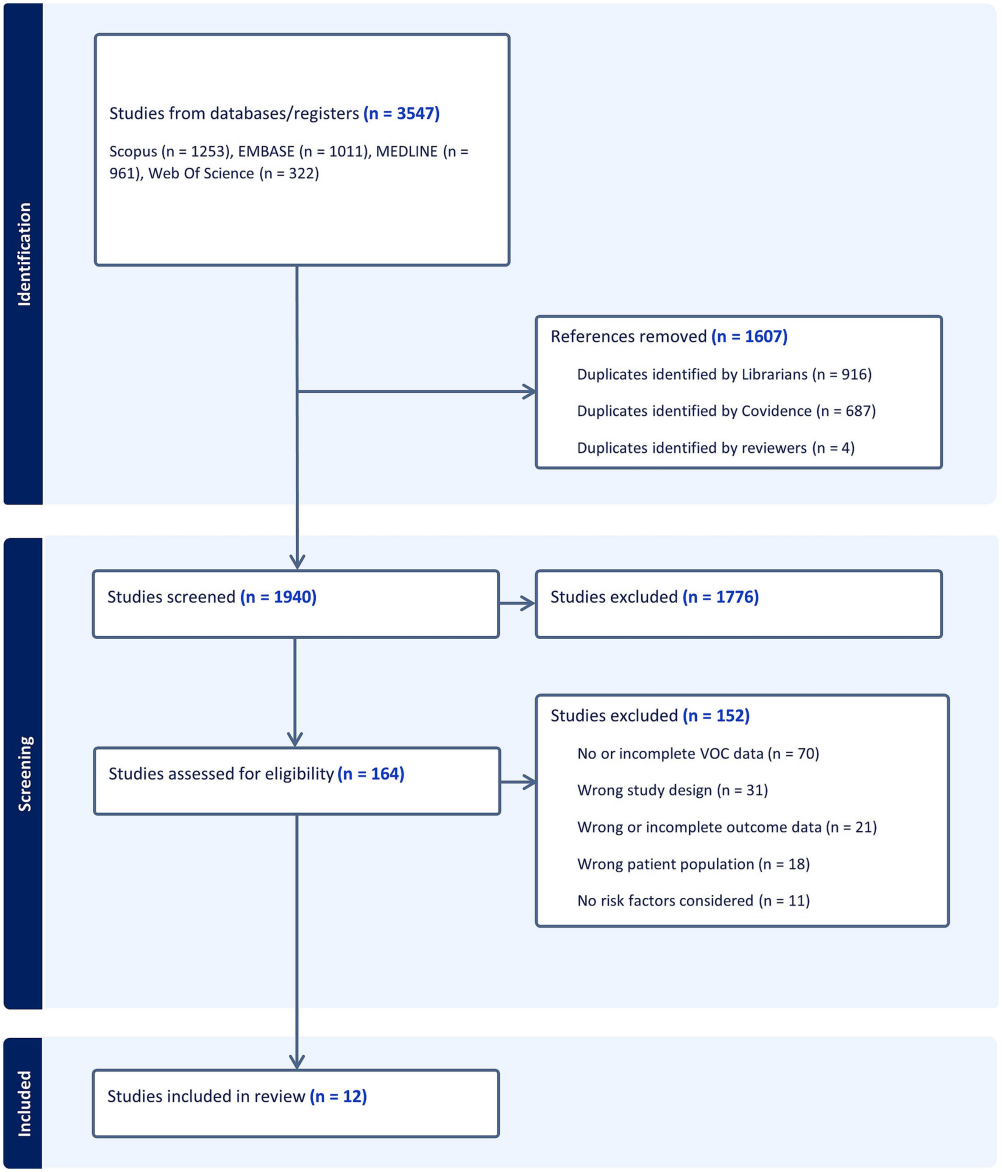
Flow-chart, adapted from PRISMA. Illustration of screening process of systematic reviews, from initial selection to the final studies included for data extraction.

We identified 3,547 articles: 1,253 from Scopus, 1,011 from Embase, 961 form PubMed and 322 from Web of Science. After deduplication, 1,940 articles underwent title and abstract screening resulting in 164 studies selected for full-text review. The entire screening process ultimately included 12 articles in the final review (Figure 1) (10–21).

### Characteristic of included studies

The characteristics of the selected systematic reviews are summarized in Table 1. Among the 12 studies included, the majority used data published prior to 2023 (8/12, ~ 67%), and conducted a meta-analysis (9/12, ~ 75%). The number of total primary studies included in each systematic reviews ranged from 5 to 92. The VOCs examined were predominantly Omicron (12/12) and Delta (11/12). Four reviews focused exclusively on Omicron and Delta infections, one systematic review compared Omicron with all previous VOCs combined as one single group and one systematic review compared two different Omicron subvariants (BA1 and BA2). The study populations ranged from 8,850 to 670,913,033.

**Table 1:** Characteristics of the 12 included studies in the overview of reviews.

First author, Year of publication	Last search	Source database	Meta-analysis	Inclusion criteria of the studies	Clinical outcomes considered	VOCs	N° of studies
Arabi et al. (20)	1st March 2022	PubMed, Medline, Embase, Scopus, Web of Science, Science Direct, MedRxiv, Lens.org	No	Omicron SARS-CoV-2 infected patients.	Hospitalization ICU admission Oxygen support IV Cardiovascular and hematological complications, Death	Omicron, Delta, Alpha, Beta, Wild Type	62
Arabi et al. (21)	6th March 2022	PubMed, Medline, Embase, Scopus, Web of Science, Science Direct, MedRxiv, Lens.org	No	Data on efficacy of previous SARS-CoV-2 infection *vs* Omicron infection.	SARS-CoV-2 reinfection or severe complications Duration of immunity	Omicron, Pre-Omicron	27
Deng et al. (19)	14th August 2022	PubMed, Embase, Web of Science, Science Direct, medRxiv, bioRxiv	Yes	Maternal or perinatal outcomes of SARS-CoV-2 infection. Virological data on SARS-CoV-2 VoCs available Epidemiological data SARS-CoV-2 VoCs. available	ICU admission Maternal death Mild or moderate disease Severe or critical disease Oxygen support NIV IV ECMO Anti-SARS-CoV-2 treatments Placental abruption PPH Blood transfusion Cesarean section Preeclampsia or Eclampsia Preterm birth <37 week Newborn SARS-CoV-2 infection Stillbirth	Omicron, Delta, Gamma, Beta, Alpha	18
Du et al. (17)	20th July 2022	PubMed, Embase, ScienceDirect	Yes	Patients hospitalized Patients with long COVID-19 Virological data on SARS-CoV-2 VoCs available	Clinical symptoms PFT Chest CT Quality of life	Alpha, Beta, Gamma, Delta, Omicron, Wild Type	51
Kow et al. (15)	7th June 2022	PubMed, Web of Science, Scopus, Google Scholar, medRxiv, Research Square, SSRN	Yes	Delta vs. Omicron SARS-CoV-2 infected patients.	Mortality severe disease ICU admission IV	Omicron, Delta	12
Hu et al. (16)	10th November 2022	PubMed, Web of Science, bioRvix, medRvix	Yes	Delta *vs* Omicron SARS-CoV-2 infected patients	Hospitalization ICU admission IV Death	Omicron, Delta	33
Nguyen et al. (14)	31th March 2023	Pubmed, Google Scholar	Yes	All adult SARS-CoV-2 re-infected patients. Virological data on SARS-CoV-2 VoCs available	Hospitalization Severe disease Clinical symptoms	Alpha, Delta, Omicron, Other	26
Relan et al. (13)	31st May 2023	WHO COVID-19 Research database	Yes	Delta *vs* Omicron SARS-CoV-2 infected patients.	Hospitalizations ICU admission Oxygen support HFNC NIV IV Death	Delta, Omicron	42
Reynolds et al. (12)	May 2023	PubMed, Medline OVID, Web of Science, CAB direct	No	Data on efficacy of COVID-19 vaccines *vs* SARS-CoV2 VoCs infections available	Hospitalization Severe disease Death	Alpha, Delta, Omicron	92
Yu et al. (10)	18th April 2022	PubMed	Yes	Delta *vs* Omicron SARS-CoV-2 infected patients	Symptomatic infection Severe disease	Omicron, Delta	68
Yuan et al. (11)	1st February 2022	PubMed/MEDLINE, Embase, Web of Science (WOS), and China National Knowledge Infrastructure (CNKI)	Yes	SARS-CoV-2 infected patients	cCHR cCFR HFR HIR	Alpha, Beta, Delta, Omicron	13
Drysdale et al. (18)	3rd November 2022	MEDLINE, Embase, LitCOVID, Cochrane COVID-19 Study Register, Econlit, ArRvix, BioRvix, ChemRvix, MedRvix, Preprints.irg, ResearchSquare, SSRN, Conference abstracts (IDWeek, ERS, ECCMID)	No	SARS-CoV-2 infected patients who received Sotrovimab during BA.1 variant predominance	Hospitalization, ICU admission, ED visit, respiratory support, COVID-19 progression, mortality	Omicron BA.1, Omicron BA.2	5

VOCs, variants of concern; SARS-CoV-2, severe acute respiratory syndrome coronavirus 2; ICU, intensive care unit; IV, invasive ventilation; HFNC, high-flow nasal cannula; NIV, non-invasive ventilation; ECMO, Extracrporeal Membrane Oxygenation; PPH, post partum hmorrhage; PFT, pulmonary function tests; CT, computed tomography; cCHR, confirmed case-hospitalization risk; cCFR, confirmed case-fatality risk; HFR, hospitalization-fatality risk; HIR, hospitalization ICU-risk; ED, emergency department.

### Clinical outcomes

Three studies compared mortality between Omicron and Delta in the overall population, and all of them reported a statistically significant lower odds or risk of mortality for patients infected with Omicron (Table 2), specifically RR 0.39 CI 95% 0.33–0.46, OR 0.33 CI 95% 0.16–0.67 (15) (4) and OR 0.34 CI 95% 0.25–0.46 (5, 6) (13, 15, 16). These results were also confirmed by the single systematic review adjusting for vaccination status (6), and by the subgroup analysis focusing on hospitalized patients and pregnant women (5, 7) (13, 16, 19). Only one review assessed the OR of mortality between Delta and pre-Delta in pregnant women, reporting lower odds in the pre-Delta era (7) (Table 2) (19). Omicron was statistically associated with lower odds and risk of ICU admission and invasive ventilation compared to Delta in the overall population and in the analyses considered, including analysis restricted to hospitalized patients and pregnant women (Table 3).

**Table 2:** Estimates of mortality associated with different VOCs, including effect sizes and mortality estimates, extracted from the selected systematic reviews.

VOCs	No of studies	No of patients	Estimates (95% CI)	Delta risk (*n*/*N*), %
Overall population				
Omicron vs. Delta (Kow, 2023)	4	70.038	OR 0.33 (0.16–0.67)	611/42.238, 1.45%
Omicron vs. Delta (Hu, 2023)	13	5.371.394	OR 0.34 (0.25–0.46)	54.044/225.189, 23.99%
Omicron vs. Delta (Relan, 2023)	18	1.285.786	RR 0.39 (0.33–0.46)	37.897/391.155, 9.69%
Adjustments				
Omicron vs. Delta, [adjusted for vaccination] (Relan, 2023)	9	1.085.771	RR 0.39 (0.32–0.47)	1.836/246.049, 0.75%
Subgroups				
Omicron vs. Delta [only hospitalized] (Hu, 2023)	8	301.309	OR 0.69 (0.58–0.82)	18.402/171.663, 10.72%
Omicron vs. Delta [only pregnant women] (Deng, 2022)	3	1.050	OR 0.22 (0.06–0.82)	127/27.736, 0.46%
pre-Delta vs. Delta [only pregnant women] (Deng, 2022)	6	119.732	OR 0.28 (0.22–0.37)	107/91.996, 0.12%

Overlap by means of GROOVE tool: Kow 2023 and Hu 2023: 16.7%; Kow 2023 and Relan 2023: 15%; Hu 2023 and Relan 2023: 18.5%; Relan 2023 and Deng 2022: 2%; Hu and Deng 2022: 0%. VOCs, variants of concerns; CI, confidence interval; *n*/*N*, total number of patients with ICU admission or invasive ventilation/overall number of patients in the delta group; OR, odds ratio; RR, risk ratio.

**Table 3:** Estimates of ICU admission associated with different VOCs, including effect sizes and mortality estimates, extracted from the selected systematic reviews.

VOCs	No of studies	No of patients	Estimates (95% CI)	Delta risk (*n*/*N*), %
*ICU admission*				
Overall population				
Omicron vs. Delta (Hu, 2023)	13	2.977.877	OR 0.27 (0.20–0.38)	57.769/1.574.082, 3.67%
Omicron vs. Delta (Relan, 2023)	15	217.561	RR 0.46 (0.37–0.57)	22.181/151.323, 14.65%
*Subgroups*				
Omicron vs. Delta [only hospitalized] (Hu, 2023)	8	301.309	OR 0.61 (0.50–0.76)	35.534/171.663, 20.69%
Omicron vs. Delta [only pregnant women] (Deng, 2022)	2	N/A	OR 0.15 (0.02–0.97)	236/27.427, 0.86%
pre-Delta vs. Delta [only pregnant women] (Deng, 2022)	5	N/A	OR 0.37 (0.21–0.67)	468/91.657, 0.51%
Invasive ventilation				
Overall population				
Omicron vs. Delta (Hu, 2023)	9	484.717	OR 0.32 (0.18–0.57)	9.377/238.610, 3.93%
Omicron vs. Delta (Relan, 2023)	11	30.852	OR 0.41 (0.29–0.57)	2.071/20.566, 10.07%
Subgroups				
Omicron vs. Delta [only hospitalized] (Hu, 2023)	7	300.234	OR 0.61 (0.47–0.78)	18.639/170.997, 10.90%
Omicron vs. Delta [only pregnant women] (Deng, 2022)	2	798	OR 0.12 (0.02–0.92)	49/27.085, 0.18%
pre-Delta vs. Delta [only pregnant women] (Deng, 2022)	3	118.034	OR 0.60 (0.42–0.84)	93/90.549, 0.10%

Overlap by means of GROOVE tool: Hu 2023 and Deng 2022: 0%; Hu 2023 and Relan 2023: 18.5%. VOCs, variants of concerns; CI, confidence interval; ICU, intensive care unit; OR, odds ratio; RR, risk ratio; N/A, not available; *n*/*N*, total number of patients with ICU admission or invasive ventilation/overall number of patients in the delta group.

Regarding hospitalization, Omicron was associated with lower odds compared to Delta. Two different reviews assessed the difference between Omicron BA1 and BA2 in the estimates of hospitalization, one adjusting for vaccination and the other for the prescription of Sotrovimab, and neither found significant differences (Supplementary Table S1) (13, 18).

However, it should be noted that in the latter review, only 5 papers were included and only one of them considered the difference in hospitalization between BA.1 and BA.2 (18).

As for severe disease, patients infected with Omicron were also less likely to develop it compared to those infected with Delta (Supplementary Table S2).

### Confidence in the results

The overall confidence in the results was rated as high in only one systematic review (5) (16). Two were rated low (7, XX), and all the remaining results were rated critically low (4, 6, 7, 9–14). Regarding the critical items, only 3/12 studies reported clearly that the review methods were established prior to the conduct of the review (item 2); 11/12 had an adequate search strategy (item 4); 7/12 did not provide details or justifications for the excluded studies (item 7); 8/12 did not use or report satisfactory techniques to assess the risk of bias of the included studies (item 9); 5/12 did not consider this in their presentation of results (item 13). Only one out of eight systematic reviews that performed a meta-analysis explicitly stated in the protocol the principles guiding the decision to execute it (item 11), and only five of them considered the risk of publication bias in their results (item 15). The scores of individual studies are summarized in Table 4.

**Table 4:** Assessment of methodological quality and confidence in the results of the included systematic reviews using the AMSTAR-2 tool (critical domains in bold).

	1	2*	3	4*	5	6	7*	8	9*	10	11*	12	13*	14	15*	16	Overall rating of quality
Arabi_1 (305), 2023	Y	**PY**	N	**PY**	Y	Y	**N**	N	**N**	N	**NA**	NA	**N**	N	**NA**	Y	Critically low
Deng, 2022	Y	**Y**	N	**PY**	Y	Y	**PY**	PY	**N**	N	**N**	Y	**Y**	Y	**Y**	Y	Critically low
Du, 2022	Y	**Y**	N	**PY**	Y	Y	**PY**	PY	**PY**	N	**N**	Y	**Y**	Y	**Y**	Y	Low
Arabi_2 (483), 2023	Y	**PY**	N	**PY**	Y	Y	**N**	N	**N**	N	**NA**	NA	**N**	N	**NA**	Y	Critically low
Kow, 2023	N	**PY**	N	**PY**	Y	Y	**N**	Y	**N**	N	**N**	N	**N**	Y	**N**	Y	Critically low
Hu, 2023	Y	**Y**	Y	**PY**	Y	Y	**Y**	PY	**Y**	N	**Y**	Y	**Y**	Y	**Y**	Y	High
Nguyen, 2023	N	**N**	N	**PY**	Y	Y	**PY**	N	**N**	Y	**N**	Y	**N**	N	**Y**	Y	Critically low
Relan, 2023	Y	**PY**	Y	**PY**	Y	Y	**N**	PY	**Y**	Y	**N**	Y	**Y**	Y	**N**	Y	Critically low
Reynolds, 2023	Y	**N**	Y	**PY**	Y	Y	**N**	PY	**N**	N	**NA**	NA	**Y**	Y	**NA**	Y	Critically low
Yuan, 2023	N	**N**	N	**N**	Y	Y	**Y**	PY	**N**	N	**N**	N	**N**	N	**N**	Y	Critically low
Yu, 2023	Y	**PY**	Y	**PY**	Y	Y	**N**	PY	**N**	Y	**N**	N	**Y**	Y	**Y**	Y	Critically low
Drysdale, 2024	Y	**PY**	N	**PY**	Y	N	**N**	PY	**Y**	Y	**NA**	NA	**Y**	Y	**NA**	Y	Low

Domains (critical domains in bold): (1) PECO components; **(2) Protocol**; (3) Study design explanation; **(4) Comprehensive search strategy**; (5) Duplicate study selection; (6) Duplicate data extraction; **(7) Details of excluded studies**; (8) Descriptions of included studies; **(9) Risk of Bias assessment**; (10) Funding source; **(11) Appropriateness of meta-analysis**; (12) Meta-analysis RoB studies; **(13) Consideration of RoB for results**; (14) Heterogeneity; **(15) Meta-analysis, publication bias**; (16) Reports conflict of interest. PECO, population-exposure-comparator-outcome; RoB, risk of bias; Y, yes; PY, partially yes; N, no; NA, not available.

### Overlap analysis

Overall, the overlap between the included reviews was generally low, with 51 out of 66 combinations showing slight overlap (<5%) (Supplementary Figure S1).

## Discussion

Our systematic review shows that individuals infected with the SARS-CoV-2 Omicron variant experience a lower risk of adverse clinical outcomes compared to those infected with the Delta variant. Additionally, the overall quality of systematic reviews comparing hospital outcomes across multiple variants was generally low, and there was relatively low overlap among the systematic reviews included in our analysis.

The widespread distribution of COVID-19 vaccines and the emergence of the less virulent SARS-CoV-2 Omicron variant have significantly altered the pandemic’s trajectory. A recent systematic review, which examined the case fatality rate (CFR) of various SARS-CoV-2 variants across different continents, confirmed that Omicron poses a lower hazard compared to other variants (22).

Additionally, the reduced severity of Omicron infections may be due to its emergence 2 years into the pandemic and 1 year after the vaccination campaign began, by which time many individuals had likely acquired natural and/or vaccine-induced immunity.

Despite Omicron’s lower mortality and other adverse outcomes compared to Delta, it remains a significant public health threat. Data indicate that Omicron’s hospitalization and mortality rates are comparable to, if not higher than, those of other endemic respiratory viruses such as influenza and respiratory syncytial virus (23, 24). Existing literature suggests that individual risk factors, such as male gender, age over 60 years, smoking, hypertension, diabetes, cancer, cerebrovascular disease, chronic kidney disease, chronic pulmonary disease, and chronic liver disease, may vary in their influence depending on VOCs, and their role on COVID-19 outcomes has been extensively studied (25).

Moreover, it is well-established that the number of comorbidities also impacts COVID-19 infection outcomes, though their influence may differ by VOCs. Specifically, a study by Piralla et al. found that ICU patients with Omicron were more likely to have three or more comorbidities compared to those with Delta, indicating that Delta infections are more aggressive and require fewer underlying conditions to become severe (26). Unfortunately, our review could not determine the differential impact of individual risk factors on clinical outcomes across VOCs.

Beyond the primary findings of our overview of reviews, one result we consider equally important yet troubling is the overall poor quality of the systematic reviews analyzed. From our perspective, systematic reviews with meta-analyses represent a powerful tool for scientific dissemination, and the rigor we expect is crucial for acquiring fundamental information. It is not surprising, though disappointing, that some of this rigor may have been compromised during the early stages of the COVID-19 pandemic. However, we believe that strictly adhering to scientifically rigorous checklists for proper systematic review methodology is a responsibility we, as researchers, owe to the common good—that is, the advancement of knowledge in our challenging scientific field. This goal should not be lost even in emergency settings like pandemics, where frameworks for evidence synthesis programs exist (27, 28). Our review identified several recurrent methodological flaws across the included studies, with some of the most relevant being the lack of information on excluded studies, insufficient risk of bias assessment, and limited consideration of bias in the interpretation of results—all of which are classified as critical domains in the AMSTAR-2 checklist. Future systematic reviews on this topic should aim to address these and other methodological weaknesses more rigorously, in order to acquire more reliable information in the future.

Some limitations must be considered. First, the focus on systematic reviews may have limited our findings by excluding individual studies. Second, most included reviews compared Omicron to Delta, while few addressed the various Omicron sublineages that currently dominate the COVID-19 landscape, and no comparisons with earlier variants were available in the selected papers. However, these findings reflect the scope and limitations of the existing literature at the time of our search, as well as the eligibility criteria applied in our review.

Third, omicron is markedly more transmissible and less virulent than previous VOCs, and underreporting remains a common issue.

Finally, our study could not assess the potential impact of other characteristics affecting clinical outcomes, such as vaccination status, type of treatment, and comorbidities; however, few of the included studies reported adjustments and subgroup analyses.

To our knowledge, this is the first overview of reviews to systematically synthesize evidence on COVID-19 clinical outcomes by variant, with particular attention to the methodological quality of the included systematic reviews.

Despite its limitations, we believe our work offers meaningful contributions, offering a valuable foundation for future research and informing public health strategies in the face of an evolving SARS-CoV-2 landscape.

## Conclusion

Our overview of reviews highlights the lower health risk posed by the Omicron variant but also reveals a lack of high-quality systematic reviews regarding the role of comorbidities and other risk factors in COVID-19 progression. More research is needed to understand how different comorbidities influence severe COVID-19, and rigorous systematic reviews are essential to fill this gap and to aid in developing targeted clinical guidelines and public health policies.

## Data Availability

The original contributions presented in the study are included in the article/Supplementary material, further inquiries can be directed to the corresponding author.

## References

[1] COVID-19 Deaths | WHO COVID-19 Dashboard. (2024). Available online at: https://data.who.int/dashboards/covid19/deaths?n=o. [Accessed 16 June, 2025].

[2] Wu F Zhao S Yu B Chen Y.-M. Wang W. Genomic characterisation and epidemiology of 2019 novel coronavirus: implications for virus origins and receptor binding. The Lancet. (2020) 395:565–74. doi: 10.1016/S0140-6736(20)30251-8, PMID: 32007145 PMC7159086

[3] Alhamlan FS Al-Qahtani AA. SARS-CoV-2 variants: genetic insights, epidemiological tracking, and implications for vaccine strategies. Int J Mol Sci. (2025) 26:1263. doi: 10.3390/ijms26031263, PMID: 39941026 PMC11818319

[4] Makarova VO Shelkov A Iliukhina A Azizyan V Dolzhikova IV Vasilieva E . Real-time PCR-based test as a research tool for the retrospective detection and identification of SARS-CoV-2 variants of concern in a sample. Int J Mol Sci. (2025) 26:1786. doi: 10.3390/ijms26051786, PMID: 40076414 PMC11898500

[5] Liu B Wang Z Lu S Qi Z Zhang Z Luan J . Monitoring reported SARS-CoV-2 variants to assess the status of COVID-19 epidemics in the low epidemic state. Sci Rep. (2025) 15:10169. doi: 10.1038/s41598-025-91308-1 40128516 PMC11933414

[6] CoVariants [Internet]. (2025). Available online at: https://covariants.org/ (Accessed June 16, 2025).

[7] Gates M Gates A Pieper D Fernandes RM Tricco AC Moher D. Reporting guideline for overviews of reviews of healthcare interventions: development of the PRIOR statement. BMJ. (2022) 378:e070849. doi: 10.1136/bmj-2022-070849 35944924 PMC9361065

[8] Shea BJ Reeves BC Wells G Thuku M Hamel C Moran J. AMSTAR 2: a critical appraisal tool for systematic reviews that include randomised or non-randomised studies of healthcare interventions, or both. BMJ. (2017) 358:j4008. doi: 10.1136/bmj.j4008, PMID: 28935701 PMC5833365

[9] Pérez-Bracchiglione J Meza N Bangdiwala SI Niño de Guzmán E Urrútia G Bonfill X. Graphical representation of overlap for OVErviews: GROOVE tool. Res Synth Methods. (2022) 13:381–8. doi: 10.1002/jrsm.1557 35278030

[10] Yu W Guo Y Zhang S Kong Y Shen Z Zhang J. Proportion of asymptomatic infection and nonsevere disease caused by SARS-CoV-2 omicron variant: a systematic review and analysis. J Med Virol. (2022) 94:5790–801. doi: 10.1002/jmv.28066, PMID: 35961786 PMC9538850

[11] Yuan Z Shao Z Ma L Guo R. Clinical severity of SARS-CoV-2 variants during COVID-19 vaccination: a systematic review and Meta-analysis. Viruses. (2023) 15:1994. doi: 10.3390/v15101994, PMID: 37896770 PMC10611048

[12] Reynolds L Dewey C Asfour G Little M. Vaccine efficacy against SARS-CoV-2 for Pfizer bio NTech, Moderna, and Astra Zeneca vaccines: a systematic review. Front Public Health. (2023) 11:1229716. doi: 10.3389/fpubh.2023.1229716, PMID: 37942238 PMC10628441

[13] Relan P Motaze NV Kothari K Askie L Le Polain O Van Kerkhove MD. Severity and outcomes of omicron variant of SARS-CoV-2 compared to Delta variant and severity of omicron sublineages: a systematic review and metanalysis. BMJ Glob Health. (2023) 8:e012328. doi: 10.1136/bmjgh-2023-012328, PMID: 37419502 PMC10347449

[14] Nguyen NN Nguyen YN Hoang VT Million M Gautret P. SARS-CoV-2 reinfection and severity of the disease: a systematic review and Meta-analysis. Viruses. (2023) 15:967. doi: 10.3390/v15040967, PMID: 37112949 PMC10145185

[15] Kow CS Ramachandram DS Hasan SS. The risk of mortality and severe illness in patients infected with the omicron variant relative to delta variant of SARS-CoV-2: a systematic review and meta-analysis. Ir J Med Sci. (2023) 192:2897–904. doi: 10.1007/s11845-022-03266-6, PMID: 36754948 PMC9908500

[16] Hu FH Jia YJ Zhao DY Fu XL Zhang WQ Tang W. Clinical outcomes of the severe acute respiratory syndrome coronavirus 2 omicron and Delta variant: systematic review and meta-analysis of 33 studies covering 6 037 144 coronavirus disease 2019–positive patients. Clin Microbiol Infect. (2023) 29:835–44. doi: 10.1016/j.cmi.2023.03.017, PMID: 36934872 PMC10023211

[17] Du M Ma Y Deng J Liu M Liu J. Comparison of long COVID-19 caused by different SARS-CoV-2 strains: a systematic review and Meta-analysis. Int J Env Res Public Health. (2022) 19:16010. doi: 10.3390/ijerph192316010, PMID: 36498103 PMC9736973

[18] Drysdale M Gibbons DC Singh M Rolland C Lavoie L Skingsley A. Real-world effectiveness of sotrovimab for the treatment of SARS-CoV-2 infection during omicron BA.2 subvariant predominance: a systematic literature review. Infection. (2024) 52:1–17. doi: 10.1007/s15010-023-02098-5, PMID: 37776474 PMC10811031

[19] Deng J Ma Y Liu Q Du M Liu M Liu J. Association of Infection with different SARS-CoV-2 variants during pregnancy with maternal and perinatal outcomes: a systematic review and Meta-analysis. Int J Env Res Public Health. (2022) 19:15932. doi: 10.3390/ijerph192315932, PMID: 36498007 PMC9740636

[20] Arabi M Al-Najjar Y Sharma O Kamal I Javed A Gohil HS. Role of previous infection with SARS-CoV-2 in protecting against omicron reinfections and severe complications of COVID-19 compared to pre-omicron variants: a systematic review. BMC Infect Dis. (2023) 23:1–16. doi: 10.1186/s12879-023-08328-3 37365490 PMC10294418

[21] Arabi M Al-Najjar Y Mhaimeed N Salameh MA Paul P AlAnni J. Severity of the omicron SARS-CoV-2 variant compared with the previous lineages: a systematic review. J Cell Mol Med. (2023) 27:1443–64. doi: 10.1111/jcmm.17747, PMID: 37203288 PMC10243162

[22] Xia Q Yang Y Wang F Huang Z Qiu W Mao A. Case fatality rates of COVID-19 during epidemic periods of variants of concern: a meta-analysis by continents. Int J Infect Dis. (2024) 141:106950. doi: 10.1016/j.ijid.2024.01.017, PMID: 38309460

[23] Grant R de Kraker MEA Buetti N Jackson H Abbas M Sobel JA . In-hospital outcomes of healthcare-associated coronavirus disease 2019 (omicron) versus healthcare-associated influenza: a retrospective, Nationwide cohort study in Switzerland. Clin Infect Dis. (2024):558. doi: 10.1093/cid/ciae558, PMID: 39535247

[24] Nørgaard SK Nielsen J Nordholm AC Richter L Chalupka A Sierra NB . Excess mortality in Europe coincides with peaks of COVID-19, influenza and respiratory syncytial virus (RSV), November 2023 to February 2024. Eurosurveillance. (2024) 29:2400178. doi: 10.2807/1560-7917.ES.2024.29.15.2400178 38606570 PMC11010589

[25] Reyna-Villasmil E Caponcello MG Maldonado N Olivares P Caroccia N Bonazzetti C . Association of Patients’ epidemiological characteristics and comorbidities with severity and related mortality risk of SARS-CoV-2 infection: results of an umbrella systematic review and Meta-analysis. Biomed Ottobre. (2022) 10:2437. doi: 10.3390/biomedicines10102437, PMID: 36289699 PMC9598435

[26] Piralla A Mojoli F Pellegrinelli L Ceriotti F Valzano A Grasselli G. Impact of SARS-CoV-2 omicron and Delta variants in patients requiring intensive care unit (ICU) admission for COVID-19, northern Italy, December 2021 to January 2022. Respir Med Res. (2023) 83:100990. doi: 10.1016/j.resmer.2023.100990, PMID: 36871459 PMC9984278

[27] Hassan Murad M Nayfeh T Urtecho Suarez M Seisa MO Abd-Rabu R Hassan Eltayeb Farah M. A framework for evidence synthesis programs to respond to a pandemic. Mayo Clin Proc. (2020) 95:1426–9. doi: 10.1016/j.mayocp.2020.05.009, PMID: 32561147 PMC7833794

[28] Brignardello-Petersen R Santesso N Guyatt GH. Systematic reviews of the literature: an introduction to current methods. Am J Epidemiol. (2025) 194:536–42. doi: 10.1093/aje/kwae232, PMID: 39038802 PMC11815505

